# Microwave Devulcanized Crumb Rubbers in Polypropylene Based Thermoplastic Dynamic Vulcanizates

**DOI:** 10.3390/polym10070767

**Published:** 2018-07-12

**Authors:** Dániel Ábel Simon, István Zoltán Halász, József Karger-Kocsis, Tamás Bárány

**Affiliations:** 1 Department of Polymer Engineering, Faculty of Mechanical Engineering, Budapest University of Technology and Economics, Műegyetem rkp. 3., H-1111 Budapest, Hungary; simond@pt.bme.hu (D.Á.S.); halaszi@pt.bme.hu (I.Z.H.); karger@pt.bme.hu (J.K.-K.); 2 MTA-BME Research Group for Composite Science and Technology, Műegyetem rkp. 3., H-1111 Budapest, Hungary

**Keywords:** thermoplastic dynamic vulcanizate, crumb rubber, ground tire rubber, rubber recycling, microwave devulcanization

## Abstract

Because of the chemically crosslinked 3D molecular structure of rubbers, their recycling is a challenging task, especially when cost efficiency is also considered. One of the most straightforward procedures is the grinding of discarded rubber products with subsequent devulcanization. The devulcanized rubber can be used as a feedstock for fresh rubber compounds or can be blended with uncured virgin rubber and thermoplastic polymers to form thermoplastic dynamic vulcanizates (TDVs). TDVs combine the beneficial (re)processability of thermoplastics and the elastic properties of rubbers. Our current work focuses on the development of polypropylene (PP)-based TDVs with the use of a tire model rubber (MR) composed of natural rubber (NR) and styrene-butadiene rubber (SBR) in a ratio of 70/30. The research target was the partial substitution of the above fresh MR by microwave devulcanized crumb rubber (dCR). TDVs were produced by continuous extrusion, and the effects of composition (PP/MR/dCR = 40/60/0…50/35/15) and processing parameters (different screw configurations, temperature profiles, the feeding method of PP) were investigated. Results showed that the fresh rubber compound can be replaced up to 10 wt % without compromising the mechanical properties of the resulting TDV.

## 1. Introduction

Polymeric materials have gained enormous importance in the last few decades. Thermoplastics and thermosets are extensively used in almost every facet of life, due to their beneficial and diversely customizable properties. However, polymeric materials pose environmental hazards and their waste management is problematic. As a result of their chemically stable structure, the decomposition of polymeric wastes in most cases is excessively slow, so polymeric products may exist for many decades after they are discarded. This problem is more substantial and acute in the field of thermoset polymers, especially with rubbers, because of their chemically crosslinked 3D molecular structure. Rubbers cannot be recycled like thermoplastics via relatively simple and cost-effective reprocessing (remelting, remolding) methods. Therefore, the development of feasible recycling technologies for rubbers attracts a great deal of academic and industrial interest. Besides other opportunities such as pyrolytic breakdown [1], another straightforward and promising route consists of grinding the used rubber products followed by their devulcanization [2,3,4]. Devulcanization is a selective breakdown of the crosslinked structure, involving the breakup of the intermolecular sulfuric crosslinks formed during the former vulcanization process [5]. Then, the devulcanized rubber can be used as a feedstock for fresh rubber compounds [4,6] or can be blended with thermoplastic polymers and fresh rubbers/curatives to form thermoplastic dynamic vulcanizates (TDVs) [7,8]. TDVs are relatively new members of the family of thermoplastic elastomers (TPEs). TPEs are thermoplastic polymeric materials combining the easy and cost-effective processability and reprocessability of thermoplastics and the elastic properties of rubbers [9,10,11]. TDVs are produced by the incorporation of rubbers into molten thermoplastics under intensive kneading/mixing, during which the rubber is selectively cured and becomes finely dispersed in the thermoplastic matrix. This “dynamic” curing (termed vulcanization when sulfuric curatives are used) leads to a fine dispersion of the partly or fully crosslinked rubber phase in the thermoplastic matrix, which is often termed as a salami or sea-island structure [12,13]. The thermoplastic “matrix” phase provides the processability and reprocessability of the melt, while the rubbery behavior can be attributed to the matrix ligaments between the rubber particles, which may undergo inhomogeneous deformations without plastic deformation (yielding) [14]. Though various thermoplastics can serve as matrices, the majority of researchers have used polyolefins (low-density polyethylene [15,16], linear low-density polyethylene [17], high-density polyethylene [8,18,19], and polypropylene (PP) [7,20]). This is due to their wide availability (eventually from recyclable waste resources) and reasonable price. Proper adhesion between the rubber and the thermoplastic phase is essential for good overall mechanical performance. Besides the utilization of various compatibilizing agents [21,22], another straightforward opportunity to enhance the mechanical performance of scrap crumb rubber-based TDVs is the incorporation of fresh rubber along with the dCR into the thermoplastic matrix [16,23,24].

This paper focuses on the effect of different processing conditions and parameters on the mechanical properties of various TDVs composed of PP, styrene-butadiene rubber (SBR)/natural rubber (NR) fresh tire model rubber blend, and microwave devulcanized crumb rubber. TDV samples were produced continuously by extrusion compounding. The effects of different screw configurations, different PP/model rubber (MR)/devulcanized crumb rubber (dCR) contents, processing temperatures, and feeding methods of the PP were studied. dCR was introduced into the TDV formulations up to 15 wt % based on the overall polymer content. Mechanical and morphological properties of the TDVs were assessed. The mechanical tests covered quasistatic tensile and tension set properties as well.

## 2. Materials and Methods

### 2.1. Materials

Random polypropylene (PP) copolymer as a thermoplastic material, natural rubber (NR) and styrene-butadiene rubber (SBR) as rubber, and waterjet-milled ground tire rubber as crumb rubber (CR) were selected and used. Manufacturers and types and basic properties of CR, rubbers, and the PP resin are listed in Table 1.

The additives of the tire model rubber (MR) formulation and their suppliers were the following: zinc oxide (ZnO 99.7%, Werco Metal, Zlatna, Romania), stearic acid (Radiacid 0444, Oleon, Ertvelde, Belgium), N550 carbon black (Omsk Carbon Group, Omsk, Russia), naphthenic oil (Tudalen 4353, H&R Group, Hamburg, Germany), N-cyclohexyl-2-benzothiazolesulfenamide (CBS, Rhenogran CBS, Rhein Chemie, Mannheim, Germany), tetramethyl thiuram disulfide (TMTD, Vulkacit Thiuram, Lanxess, Cologne, Germany), and sulphur (Powder Sulphur, Astrakhan, Russia).

### 2.2. Processing Techniques and Parameters

Microwave devulcanization of the CR was carried out in a BP-125/50 type laboratory microwave oven, produced by Microwave Research Inc. (Carol Stream, IL, USA) with batch sizes of 100 g. The final temperature was fixed at 200 °C and the heating rate was 6 °C/min. Prior to microwave treatment, the CR was kept at 150 °C for 2 h in a Venticell LSIS-B2V/VC55 hot air oven (MMM Group, Monroe, WA, USA). The microwaving of the CR resulted in a significant increment in its sol content. Sol content was determined by Soxhlet extraction in toluene with an extraction time of 16 h. Untreated CR and microwave-treated CRs had sol contents of 11.0 ± 1.6 wt % and 28.5 ± 1.3 wt %, respectively.

**Figure 1:**
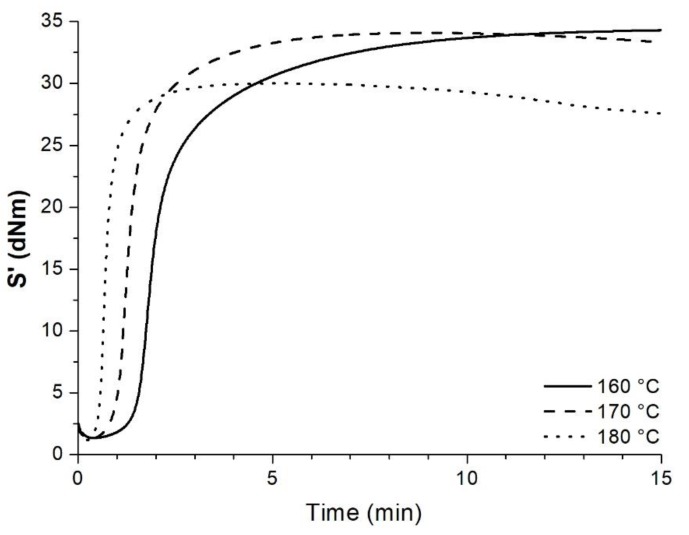
Vulcanization curves of the MR at different temperatures.

The formulation of the model rubber (MR) was: SBR 70 phr, NR 30 phr, ZnO 5 phr, stearic acid 1 phr, naphthenic oil 30 phr, N550 carbon black 60 phr, CBS 1.5 phr, TMTD 1.5 phr, sulphur 1.5 phr. The rubber ingredients were compounded by Gumiplast Ltd (Nyírmada, Hungary) in a Banbury-type internal mixer. The curing curves of MR at 160, 170, and 180 °C (1.67 Hz and 1° amplitude, measured on a MonTech D-RPA 3000 Dynamic Rubber Process Analyzer (MonTech, Buchen, Germany)) are shown in Figure 1.

The curing parameters determined from the curves are listed in Table 2.

The applicability of the dCR and the effect of its content in TDV production were studied by partial substitution of the MR by dCR in 10, 20, and 30 wt % ratios. The component formulations of the TDVs are shown in Table 3.

The compounding and dynamic vulcanization of the blends were performed on a modular corotating twin-screw extruder (Labtech LTE 26-44, Labtech Engineering Co. Ltd., Samutprakarn, Thailand) with a screw speed of 180 rpm and two different temperature profiles. The temperature profiles of the twin-screw extruder are displayed in Table 4.

Prior to compounding and dynamic vulcanization, the rubber mix was shaped on a single-screw extruder (Labtech 25-30 C, Labtech Engineering Co. Ltd., Samutprakarn, Thailand, 40 rpm screw speed and 95–105 °C zone temperatures), and a single rubber filament was obtained with a diameter of 3 mm to ensure the precise and reproducible dosing of the rubber during TDV production. The rubber was dosed by feeding the rubber filament continuously into the hopper of the twin-screw extruder. PP was fed in two different ways so that effects of different feeding methods could be studied: (i) feeding the PP with the rubber filament through the hopper (H) of the twin-screw extruder simultaneously, and (ii) dosing PP in melted state into the second zone of the twin-screw extruder with the help of a single-screw extruder (Labtech 25-30 C, Labtech Engineering Co. Ltd., Samutprakarn, Thailand, *D* = 25 mm, *L*/*D* = 30) and a side feeder (S) adaptor. The given MR/PP ratio was set by the following method: net uncured rubber intake at the given screw speed of the twin-screw extruder (180 rpm) was measured (4.5 kg/h), and the necessary amount of additional PP was calculated and set by (i) utilizing and adjusting the built-in hopper feeder of the twin-screw extruder to grant 4.5 kg/h feeding of PP granules (in case of hopper PP feeding and 50/50 MR/PP ratio), or (ii) adjusting the screw speed of the side feeder of the single-screw extruder to obtain 4.5 kg/h (50/50 PP/MR ratio) and 3 kg/h (40/60 PP/MR ratio) throughput for the neat PP melt (in case of side feeding). Three different temperature profiles were set on the single-screw extruder, which are summarized in Table 5. The screw speed of the side feeding extruder was set according to the PP and rubber ratios of the various formulations (cf. data in Table 3.).

**Figure 2:**
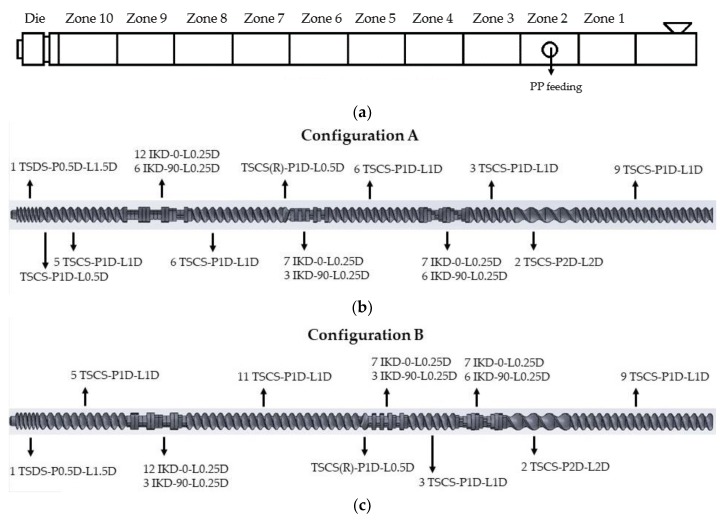
Extruder zones and screw configurations: (**a**) Location of the zones along the extruder; (**b**) Screw configuration A; (**c**) Screw configuration B. Designations of the screw modules (*D* stands for the diameter of the screw): TSCS-P1D-L1D: twin-start conveying screw, pitch 1*D*, length 1*D*; TSCS(R)-P1D-L1D: twin-start conveying screw (reverse flight), pitch 1*D*, length 1*D*; TSCS-P2D-L2D: twin-start conveying screw, pitch 2*D*, length 2*D*; IKD-0-L0.25D: individual kneading disc, length 0.25*D*, 0° angle; IKD-90-L0.25D: individual kneading disc, length 0.25*D*, 90° angle (each kneading disc can be rotated and positioned in 60° steps on the screw, so utilizing a 0° and a 90° kneading element allowed us to build kneading blocks with individual discs having a 30° angle difference compared to the neighboring blocks: 0°, 60°, 120°; 180°; 240° and 300° was achieved with discs with a 0° angle, while 30°, 90°, 150°, 210°, 270° and 330° was achieved with discs with a 90° angle); TSDS-P0.5D-L1.5D: twin-start discharge screw, pitch 0.5*D*, length 1.5*D*.

Effects of different screw configurations was also investigated: the zones of the twin-screw extruder and the different screw configurations are depicted in Figure 2. It can be seen that two of the three kneading blocks on screw “B” are positioned closer to the PP feeding zone and to the hopper. The concept behind it was to achieve intensive mixing and kneading at an early stage of the curing of the rubber phase, which may lead to finer dispersion and an improvement in the mechanical performance of the resulting TDVs. At the middle kneading block, a conveying element with reverse flight was implemented to decelerate the melt flow and increase the pressure. This way, the preceding kneading block kneaded/sheared the compound even more intensively. Conveying elements with double length and pitch (TSCS-P2D-L2D) were selected for the PP feeding zone to produce the necessary free volume for the PP melt. At the end of the screw, a conveying element with 1.5*D* length and 0.5*D* pitch was used to increase the pressure for more homogenous and more uniform output.

The extruded TDV filaments were cooled by air and pelletized (Labtech LZ-120/VS pelletizer, Labtech Engineering Co. Ltd., Samutprakarn, Thailand) after compounding. From the pellets, ISO 3167 A type dumb-bell specimens were injection-molded with an Arburg Allround Advance 370S 700-290 injection-molding machine (Arburg Ltd., Lossburg, Germany). The related parameters were: barrel temperature range: 170–190 °C, mold temperature: 30 °C, injection speed: 50 cm^3^/s, shot volume: 44 cm^3^, and holding pressure: 350 bar. Abbreviations and processing parameters of the TDV samples are summarized in Table 6.

### 2.3. Testing Methods and Parameters

Hardness was tested according to the ISO 868 Shore D method on a Zwick H04.3150.000 hardness tester (Zwick GmbH., Ulm, Germany) on the injection-molded specimens. Ten tests were performed on each compound, followed by the calculation of average and standard deviation values.

The tensile mechanical properties of the compounds were investigated according to the ISO 527 standard on a Zwick Z250 universal testing machine with a 20 kN load cell (Zwick GmbH., Ulm, Germany) at room temperature at a 100 mm/min crosshead speed. This deformation rate, being below that of the typical rubber testing (500 mm/min), was selected to compare the tensile performance of the TDVs with the PP matrix. The average and standard deviation of the tensile strength and elongation at break were determined with 5 tests on each compound.

The tension set of the investigated compounds was tested on a Zwick Z020 universal testing machine with a 20 kN load cell (Zwick GmbH., Ulm, Germany) at room temperature with a clamping length of 110 mm. A test length of 70 mm was marked on the narrow section of the specimen by two markers, then the specimen was stretched until clamping length reached 180 mm. Specimens were kept stretched for 24 h at room temperature, then unloaded and the distance between the two markers were measured after 0, 5, 1, 3, 6, 12 and 24 h after the removal of the load. Tension set (%) values were calculated with Equation (1).

(1) $$\;TS=\frac{L_{2}-L_{0}}{L_{1}-L_{0}}\cdot 100\;\;\;\;$$

where *L*_0_ (mm) is the initial test length (*L*_0_ = 70 mm), *L*_1_ (mm) is the stretched distance between the markers, and *L*_2_ (mm) is the distance between the markers after the given relaxation time.

The morphology of the TDVs was characterized with SEM micrographs. SEM images of cut surfaces produced by cryogenic microtome were taken with a Jeol JSM 6380 LA scanning electron microscope (Jeol Ltd., Tokyo, Japan). Prior to investigation, the surfaces of the specimens were sputter-coated with gold.

## 3. Results and Discussion

### 3.1. Mechanical Properties

**Figure 3:**
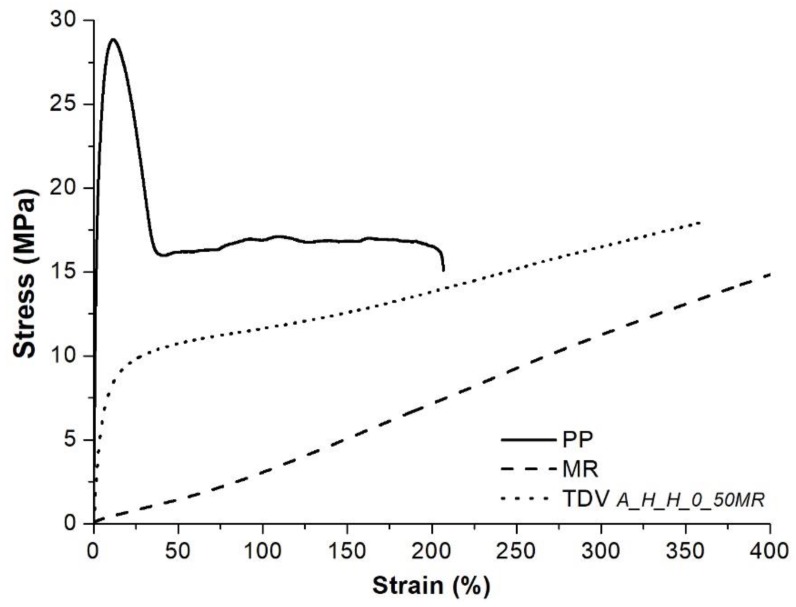
Characteristic tensile curves of the PP, MR, and one of the TDVs.

Characteristic tensile curves of the PP, the cured MR (cured at 180 °C), and the “A_H_H_0_50 MR” TDV are shown in Figure 3. It can be seen that the TDV has a higher initial modulus compared to the MR, but after around 25% elongation, a rubber-like, low modulus deformation can be observed.

**Figure 4:**
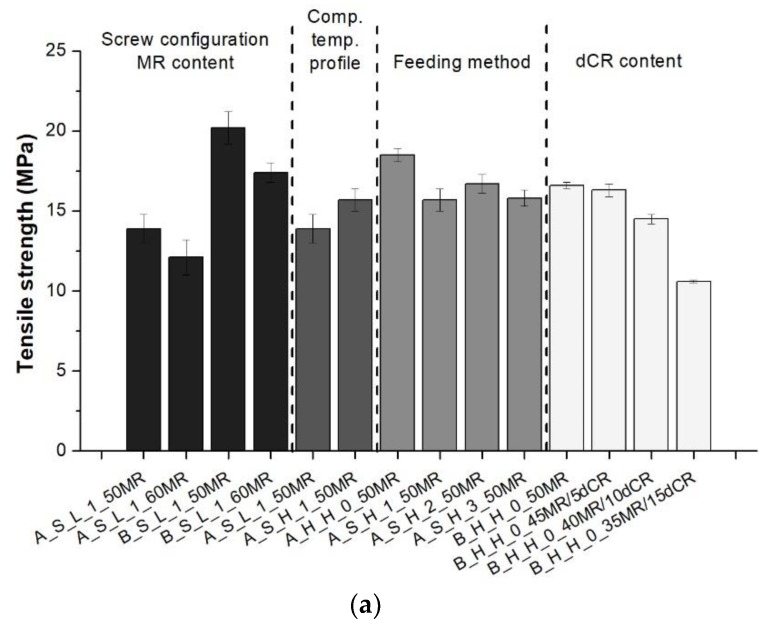

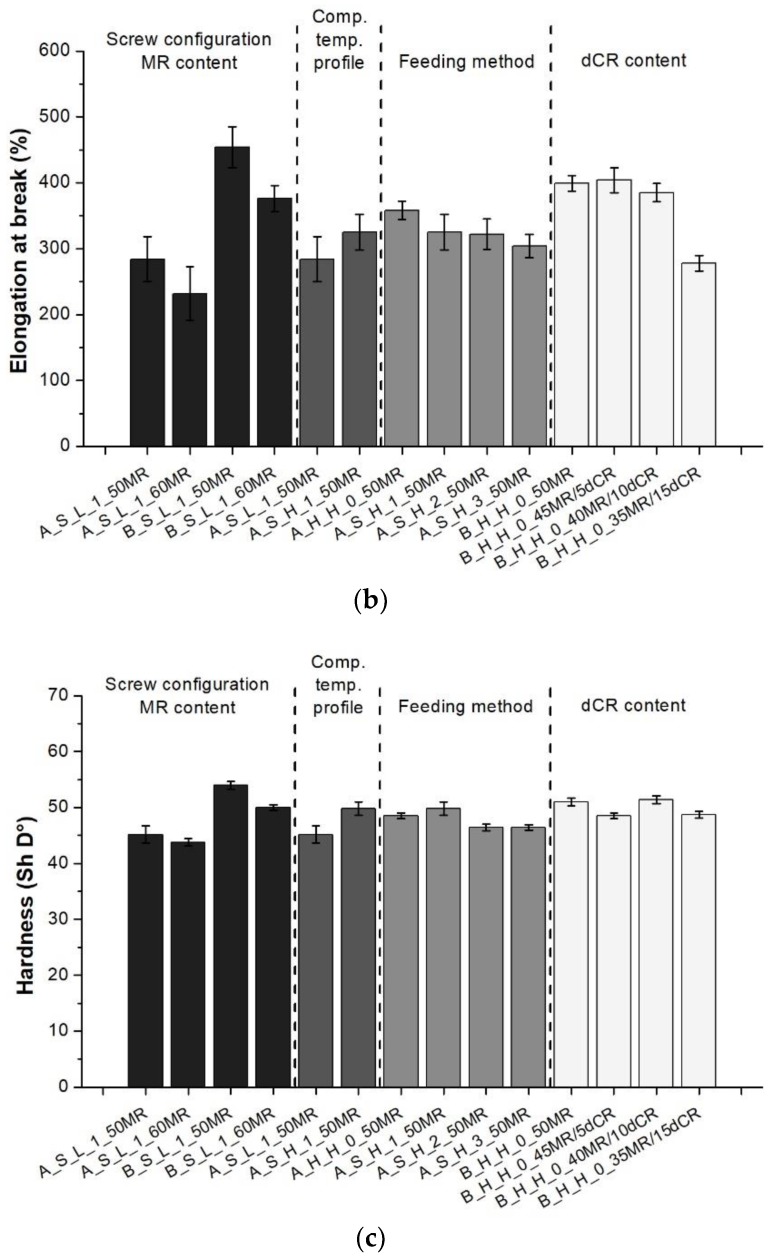
Ultimate tensile properties and hardness of the produced TDVs: (**a**) Tensile stress; (**b**) Elongation at break; (**c**) Hardness.

The results of the mechanical tests were arranged in four categories based on the various variables studied, namely (i) screw configuration and MR content; (ii) the temperature profile of the compounding twin-screw extruder; (iii) PP feeding method; (iv) dCR content. The tensile strength, elongation at break, and hardness values of the produced TDVs are summarized in Figure 4; stresses at 100%, 200% and 300% elongation and tension set results are displayed in Section 3.1.1, Section 3.1.2, Section 3.1.3 and Section 3.1.4, respectively.

#### 3.1.1. Effects of Screw Configurations and MR Content

**Figure 5:**
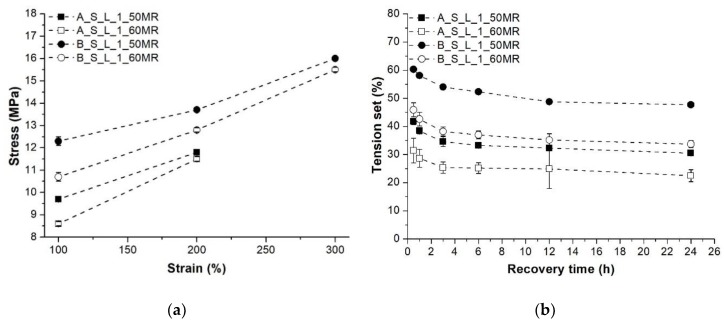
Stress at 100%, 200% and 300% elongation and the tension set of the TDVs produced with different screw configuration and MR content: (**a**) Stress; (**b**) Tension set.

Similar tendencies can be observed regarding the tensile stress and elongation at break of the investigated compounds. Screw configuration “B” and lower MR content resulted in improved tensile properties (Figure 4a,b). Figure 5 depicts the stress values measured at 100%, 200% and 300% elongation, and the tension set data of the TDVs produced with different screw configuration and MR content. Screw configuration “A” led to higher stresses at the observed elongations (TDVs produced with screw configuration “B” in this series had elongation at break values less than 300%, therefore stress at 300% elongation could not be presented), and increasing MR content resulted in a slight decrease in the recorded values, in line with our expectations. Tension set values nevertheless showed an opposite tendency regarding screw configuration. TDVs produced with screw configuration “A” performed reasonably better, having considerably higher elastic recovery. Increasing MR content predictably brought higher recovery, namely lower tension set values.

#### 3.1.2. Effects of the Compounding Temperature Profile

**Figure 6:**
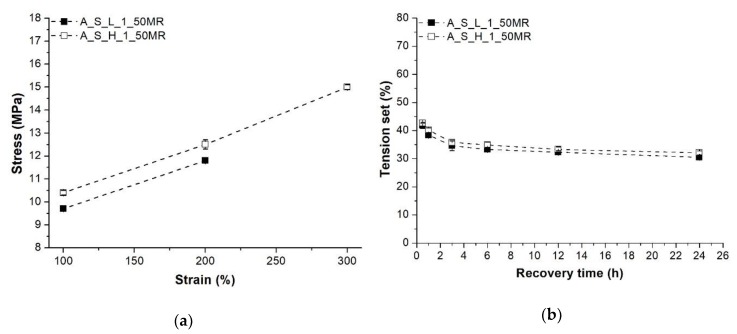
Stress at 100%, 200% and 300% elongation and the tension set of the TDVs produced with different compounding temperature profiles: (**a**) Stress; (**b**) Tension set.

Higher initial compounding temperature (compounding temperature profile “H”) resulted in improved tensile properties, and the same can be observed in terms of hardness (Figure 4). Figure 6 shows the aforementioned stress and tension set of the TDVs produced with different compounding temperature profiles. Based on these results, it can be stated that increasing the temperature of the initial extruder zones yielded not only improved ultimate tensile properties, but enhanced the stresses at given elongations (i.e., at 100%, 200%, 300%) as well. This may be attributed to the faster curing of the rubber phase, which led to decreased deformability sooner during the dynamic vulcanization, thus facilitating the disintegration of the rubber droplets. On the other hand, this variable has no significant effect on the tension set results. TDVs produced with temperature profile “L” in this series had elongation at break values less than 300%, and therefore stress at 300% elongation could not be presented.

#### 3.1.3. Effects of the Feeding Method of PP

**Figure 7:**
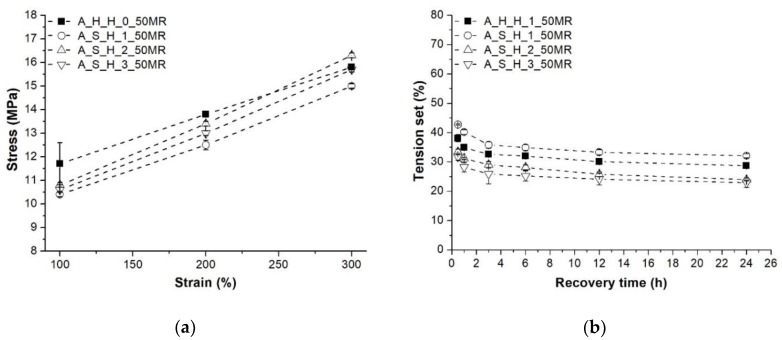
Stress at 100%, 200% and 300% elongation and the tension set of the TDVs produced with different PP feeding methods: (**a**) Stress; (**b**) Tension set.

Feeding the PP at the hopper in the form of granules had a positive effect on the tensile mechanical properties, similar to the higher initial compounding temperature. The melt temperature of the PP in the case of PP side feeding did not have a significant effect on the tensile properties (Figure 4). Stresses at given elongations and the tension set of the TDVs produced with different PP feeding methods are presented in Figure 7. Hopper feeding results in higher stresses at 100% and 200% elongations, but leads to slightly lower stress at 300% than side melt feeding with the middle temperature profile. The tension set results show that the side melt feeding of PP can improve elastic recovery. This is suggested by the results belonging to the side extruder temperature profiles “2” and ”3”.

#### 3.1.4. Effects of dCR Content

**Figure 8:**
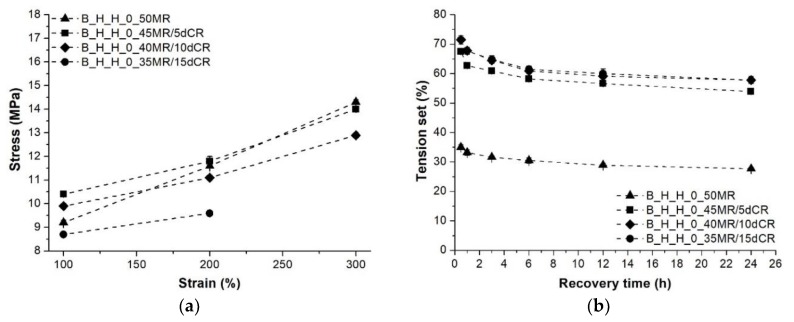
Stress at 100%, 200% and 300% elongation and the tension set of the TDVs produced with different dCR content: (**a**) Stress; (**b**) Tension set.

The processing parameters of TDVs with incorporated dCR were defined by considering the results of the tensile tests of the TDVs containing MR. Therefore, screw configuration “B”, hopper feeding of the PP, and a higher compounding temperature profile were used in the production of TDVs containing dCR. Replacing 10, 20 and 30 wt % of the MR with dCR resulted in slightly deteriorated tensile behavior; nevertheless, at 10 and 20 wt % ratios, the decline was very modest. Hardness remained practically unchanged by dCR content (cf. Figure 4). The possible reason behind this finding is that the Shore hardness of dCR was closely matched with that of the resulting TDV in this case. Tension set and stresses at 100, 200 and 300% elongations of the TDVs produced with different dCR content can be seen in Figure 8. Unfortunately, both the stress and elastic recovery of the material deteriorated when dCR was introduced into the TDV.

### 3.2. Morphology

**Figure 9:**
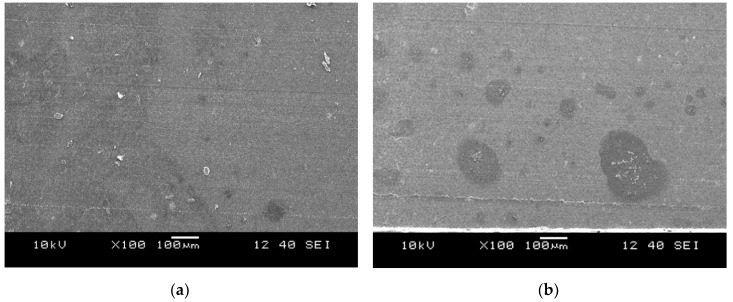
Cryomicrotomed surfaces of the TDVs produced with different screw configurations: (**a**) Screw configuration “A” (A_S_L_1_50 MR); (**b**) Screw configuration “B” (B_S_L_1_50 MR).

Figure 9 shows typical micrographs from TDVs produced with 50 wt % MR content and different screw configurations. Darker areas belong to the MR, while lighter areas to the PP phase.

According to the SEM images, screw configuration “B” resulted in better dispersion of the rubber phase, displaying rubber “islands” with small diameter. By contrast, TDVs produced with screw configuration “A” showed a coarser rubber dispersion. At low magnification, even hints for local co-continuity can be found. This can be attributed to lower shear stresses, evolved during compounding with the “A” compared to the “B” screw that delayed the dispersion of rubber (transition to droplets).

**Figure 10:**
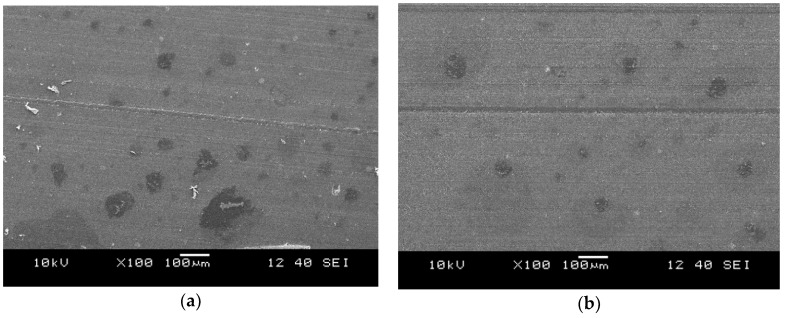
Cryomicrotomed surfaces of the TDVs with different PP feeding methods and higher compounding temperature profile: (**a**) Side feeding with 180 °C melt temperature (A_S_H_2_50 MR); (**b**) Hopper feeding (A_H_H_0_50 MR).

Figure 10 shows typical records from TDVs produced with 50 wt % MR content and different PP feeding methods.

**Figure 11:**
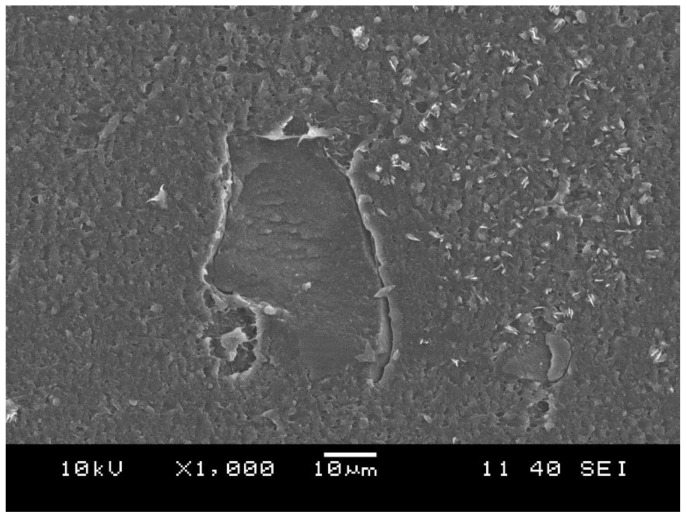
Cryomicrotomed surfaces of the TDV with 10 wt % dCR in the rubber phase.

The comparison of the images displayed in Figure 9 and Figure 10 shows that increasing the compounding temperature (Figure 9a: compounding temperature profile: L; Figure 10a: compounding temperature profile: H) resulted in a refinement of the rubber dispersion similar to the modification of the screw configuration. Hopper feeding of the PP had a similar, but additional refining effect on the dispersion of the rubber in the TDV. This may be caused by the rapid curing of the rubber phase at the zone, where the PP was introduced to the compounding extruder in the case of side feeding. This rapid vulcanization can be explained by a thermal shock “suffered” by the rubber when merging with the PP melt coming from the side feeding extruder. Fast curing could hinder the rupture of the rubber particles, and thus the dispersion of the rubber phase. Figure 11 shows a micrograph taken from the surface of a TDV containing 10 wt % dCR in the rubber phase. TDVs with the tested dCR contents had similar rubber particles having seemingly poor adhesion to the embedding matrix. This is the most reasonable explanation for decreasing tensile strength and elongation at break for the related TDVs, especially at higher dCR contents.

## 4. Conclusions

This work was aimed at studying effects of TDV formulation and processing parameters on the mechanical and morphological performance of PP-based TDVs with an SBR/NR rubber phase. Special attention was given to check whether the fresh rubber can be partially replaced by microwave devulcanized crumb rubber (dCR). Note that the use of dCR is an ecologically driven opportunity for rubber waste management. Our results showed that in terms of rubber dispersion and tensile mechanical behavior, it is highly beneficial to subject the compound to intensive mixing/kneading in a relatively early stage of the curing of the rubber phase. This can be achieved with suitable kneading elements in the initial sections of the extruder screw. Feeding both the PP and the rubber into the hopper of the extruder has a positive effect on the dispersion of rubber and thus also on mechanical properties. In this feeding, the melting of the PP occurs in the presence of the rubber compound, thereby generating higher shear stresses within the PP melt, which finally leads to a better dispersion of the rubber particles. Partial substitution of the fresh rubber compound with dCR is possible up to 10 wt % without compromising the tensile mechanical properties of the resulting TDV. Replacing 30 wt % of the fresh rubber, however, led to a pronounced drop in mechanical properties. No additional curatives were added to the dCR-containing rubber compounds in this project, because our goal was to check the effect of “pristine” dCR as a potential substituent of fresh rubbers in TDVs. According to the preliminary results, the incorporation of additional curatives into the compounds containing dCR may improve the tensile mechanical properties of the corresponding TDVs, and thus an even higher amount of dCR can be incorporated without the deterioration of properties.

## Figures and Tables

**Table 1:** Types and producers of raw materials.

Abbreviation	CR	SBR	NR	PP
**Type, Manufacturer,**	Aquajet Ltd., Budapest, Hungary	SBR 1502, SKS-30 ARKPN, JSC Sterlitamak Petrochemical Plant Sterlitamak, Russia	NR TSR 10 Sud Comoe Caoutchuc, Aboisso, Ivory Coast	TIPPLEN R 959 A MOL Petrochemicals Co. Ltd., Budapest, Hungary
**Main properties**	Waterjet-milled truck tire tread, particle size between 200 and 400 μm	Mooney viscosity (ML, 1 + 4, 100 °C): 48–58 Bound styrene content: 22–25 wt %	N/A	Random copolymer for injection molding, MFR (230 °C, 2.16 kg): 45 g/10 min, Tensile strength at yield: 30 MPa, Tensile strain at yield: 12%

**Table 2:** Curing parameters of the MR at 160, 170, and 180 °C.

Designation	*M_L_* (dNm)	*M_H_* (dNm)	*t*_10_ (min)	*t*_70_ (min)	*t*_90_ (min)
160 °C	1.36	34.35	1.53	2.57	5.37
170 °C	1.29	34.08	0.98	1.58	2.85
180 °C	1.20	30.01	0.54	0.83	1.33

**Table 3:** Abbreviations and formulation of the TDVs produced (amounts are shown in wt %).

Designation	60 MR	50 MR	45 MR/5 dCR	40 MR/10 dCR	35 MR/15 dCR
PP	40	50	50	50	50
MR	60	50	45	40	35
dCR	0	0	5	10	15

**Table 4:** Temperature profiles of the twin-screw extruder (temperature values are shown in °C).

Designation	Zones										
	1	2	3	4	5	6	7	8	9	10	Die
L	160	160	160	165	165	170	170	170	175	175	180
H	170	170	170	175	175	175	180	180	180	180	180

**Table 5:** Temperature profiles of the single-screw extruder used for side dosing of the PP (temperature values are shown in °C).

Designation	Zones				
	1	2	3	4	Adapter
1	140	145	155	160	160
2	165	170	175	108	180
3	185	190	195	200	200

**Table 6:** Abbreviations used for the TDV samples having different compositions and produced with various processing parameters.

Abbreviation	Screw configuration	PP feeding method	Compounding extruder temperature profile	PP feeding side extruder temperature profile	TDV formulation
A_S_L_1_50 MR	A	side, melt	L	1	50 MR
A_S_L_1_60 MR	A	side, melt	L	1	60 MR
B_S_L_1_50 MR	B	side, melt	L	1	50 MR
B_S_L_1_60 MR	B	side, melt	L	1	60 MR
A_S_L_1_50 MR	A	side, melt	L	1	50 MR
A_S_H_1_50 MR	A	side, melt	H	1	50 MR
A_H_H_0_50 MR	A	hopper, granules	H	(PP was fed at the hopper)	50 MR
A_S_H_1_50 MR	A	side, melt	H	1	50 MR
A_S_H_2_50 MR	A	side, melt	H	2	50 MR
A_S_H_3_50 MR	A	side, melt	H	3	50 MR
B_H_H_0_50 MR	B	side, melt	L	1	50 MR
B_H_H_0_45 MR/5 dCR	B	side, melt	L	1	45 MR/5 dCR
B_H_H_0_40 MR/10 dCR	B	side, melt	L	1	40 MR/10 dCR
B_H_H_0_35 MR/15 CR	B	side, melt	L	1	35 MR/15 dCR
